# Reproducible Molecularly Imprinted Piezoelectric Sensor for Accurate and Sensitive Detection of Ractopamine in Swine and Feed Products

**DOI:** 10.3390/s18061870

**Published:** 2018-06-07

**Authors:** Mingfei Pan, Rui Li, Leling Xu, Jingying Yang, Xiaoyuan Cui, Shuo Wang

**Affiliations:** Key Laboratory of Food Nutrition and Safety, Ministry of Education of China, Tianjin University of Science and Technology, Tianjin 300457, China; panmf2012@tust.edu.cn (M.P.); singutosleep@126.com (R.L.); apple2ringo@126.com (L.X.); Yangjy0823@126.com (J.Y.); XY.Cui@163.com (X.C.)

**Keywords:** ractopamine, molecularly imprinted piezoelectric sensor, swine and feed samples, quantitative detection

## Abstract

This paper describes the development of a reproducible molecularly imprinted piezoelectric sensor for the accurate and sensitive detection of ractopamine (RAC) in swine and feed products. The synthesized molecularly imprinted polymer (MIP) was directly immobilized on the surface of a quartz crystal microbalance (QCM) Au chip as the recognition element. The experimental parameters in the fabrication, measurement and regeneration process were evaluated in detail to produce an MIP-based piezoelectric sensor with high sensing capability. The developed piezoelectric sensor was verified to perform favorably in the RAC analysis of swine and feed products, with acceptable accuracy (recovery: 75.9–93.3%), precision [relative standard deviation (n = 3): 2.3–6.4%], and sensitivity [limit of detection: 0.46 ng g^−1^ (swine) and 0.38 ng g^−1^ (feed)]. This portable MIP-based chip for the piezoelectric sensing of RAC could be reused for at least 30 cycles and easily stored for a long time. These results demonstrated that the developed MIP-based piezoelectric sensor presents an accurate, sensitive and cost-effective method for the quantitative detection of RAC in complex samples. This research offers a promising strategy for the development of novel effective devices used for use in food safety analysis.

## 1. Introduction

Ractopamine (RAC) is an artificially synthesized β-adrenergic agonist, which was originally used as a bronchial dilating agent for the treatment of pulmonary disease and asthma [1,2]. However, in the farming industry, RAC is also illegally applied as a nutrient repartitioning agent to increase muscle and reduce fat accumulation [3,4,5]. It has been proven that RAC can be retained in animals and subsequently enters the human body through the food chain, causing cumulative toxicity. Thus, when the accumulated RAC dose exceeds a certain level, toxic reactions, such as triggering muscle tremor, tachycardia, and muscle pain, may occur [6,7,8]. To avoid the potential risk of RAC to human health, many countries (including the USA, China and the European Union) and organizations have set strict regulations for RAC and other β-adrenergic agonists, with zero tolerance in animal feed or processing [9,10]. However, RAC is still frequently detected in swine and feed products. Therefore, the development of an effective method for RAC determination to control the amount of RAC in complex samples, especially the swine and feed products, is necessary for the assurance of consumer health [11].

Currently, RAC is commonly determined via high performance liquid chromatography (HPLC) [12,13], liquid chromatography/gas chromatography-tandem mass spectrometry (LC/GC-MS) [14,15,16], enzyme-linked/fluorescence immunoassay [17], and biosensor analysis [18,19,20,21]. Among these, instrumental analysis based on the principles of chromatography and mass spectroscopy is still the main method for RAC detection. These methods are effective and reliable and can detect the presence of the target analyte (RAC) at trace levels in various food samples after a relatively simple pre-treatment process. Molecularly imprinted polymers (MIPs), also known as artificial antibodies, are synthetic materials that can specifically and selectively recognize the target analyte from various samples in a relatively harsh environment [22,23,24]. Over the past ten years, more in-depth studies on MIP synthesis and its applications have been carried out in the fields of drug delivery, food science, environmental monitoring, and medical testing. This has led to significant progress in the area with good potential for future application [25]. Due to their selective binding affinity, one of the most valuable applications of MIP is their use as solid phase extraction (SPE) sorbents for the purification and enrichment of trace substances present in complex matrices [26,27]. To date, various target analytes, such as proteins, carbohydrates, and small molecules, can be enriched as MIP binding targets. In our previous study, we prepared an RAC imprinted material with good capability. This material was further applied as an enrichment sorbent for the determination of trace RAC in pork samples using on-line solid-phase extraction coupled with HPLC. Another remarkable MIP application was its use as a recognition element in chemical sensors with different types of signals (fluorescence, impedance, current and potential signals). These sensors were stable and selective in harsh environments with low interference [28,29,30]. However, the high cross-linking degree of molecularly imprinted systems is not conducive to the transmission of electrons and photon. This has led to the reduction in sensitivity and accuracy for the detection of trace target analytes by molecularly imprinted electrochemical and photochemical sensors. Molecularly imprinted piezoelectric sensors that monitor the mass signal combine the merits of molecular imprinting and piezoelectric sensing techniques. This technique displays unparalleled advantages in the identification of signal conversion and detection sensitivity and can realize the real-time and portable analysis of different analytes [31,32,33]. Quartz crystal microbalances (QCM) are widely used as piezoelectric sensors and can effectively reflect the amount of target analyte bound on the surface of a chip by monitoring the change in the resonant frequency of the quartz crystal [34,35]. Recent years have seen interesting studies on the application of molecularly imprinted QCM sensors for the qualitative and quantitative analyses of different analytes in various fields [36,37,38,39].

**Scheme 1:**
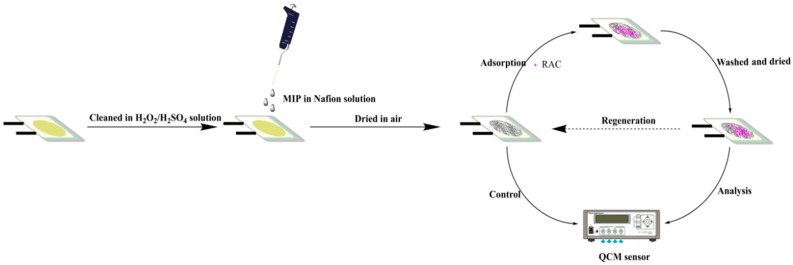
Schematic diagram of the MIP piezoelectric chip for RAC detection.

In this study, a molecularly imprinted piezoelectric sensor was constructed by directly modifying an MIP material with good recognition ability toward RAC onto the surface of a QCM chip. The sensor was further applied to detect trace RAC in swine and feed products (Scheme 1). The proposed piezoelectric sensor afforded consistent detection results via a typical HPLC-MS/MS method with a correlation coefficient (r^2^) of 0.9611, good accuracy (recovery: 75.9–93.3%), acceptable precision [relative standard deviation (n = 3): 2.3–6.4%], and high sensitivity [detection limit: 0.46 ng g^−1^ (swine) and 0.38 ng g^−1^ (feed)] in the selected samples spiked with the same levels of RAC. Furthermore, this MIP-based piezoelectric sensor exhibited several advantages such as low-cost, a short detection period, and good reproducibility. Thus, this sensor shows good potential for application as an effective device for the accurate, sensitive and cost-effective detection of RAC. This study has provided an interesting combination of molecular imprinting and piezoelectric sensing techniques as well as a new strategy for the determination of trace amounts of harmful substances in complex samples.

## 2. Materials and Methods

### 2.1. Reagents and Materials

**Figure 1:**
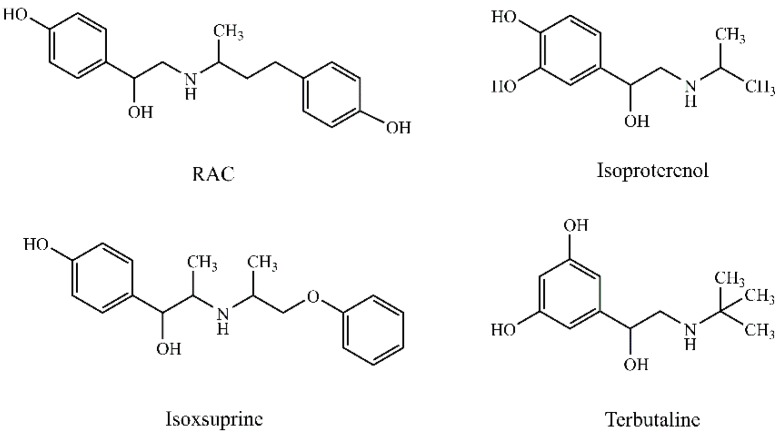
Chemical structure of RAC, isoproterenol, terbutaline and isoxsuprine.

The standard product of RAC (≥99.0%) and its analogues including isoproterenol, isoxsuprine and terbutaline (≥99.0%; Figure 1) were purchased from Sigma-Aldrich (St. Louis, MO, USA). The silane reagents 3-aminopropyltriethoxysilane (APTES) and tetraethoxysilane (TEOS) acquired from Hubei Wuhan University Silicone New Material Co. Ltd. (Wuhan, China) were used as the functional monomer and cross-linker for MIP synthesis, respectively. A Nafion^®^ perfluorinated resin solution [5 wt % in the solution of low aliphatic alcohol (55%) and water (45%), Sigma-Aldrich (St. Louis, MO, USA)] was employed for MIP immobilization on the piezoelectric chip surface. Doubly deionized water (18.2 MΩ cm) produced by a Water Pro water system (Labconco, Kansas City, MO, USA) was used throughout the whole study. All other chemicals and reagents such as methanol, acetic acid, HCl, NH_3_·H_2_O and phosphates were at least of analytical grade and purchased from Tianjin Chemical Reagent Factory (Tianjin, China).

The individual stock solutions for all the analytes (RAC, isoproterenol, isoxsuprine, terbutaline) at a concentration of 100.0 mg L^−1^ were initially prepared by dissolving 10.0 mg standard product into methanol and subsequently stored at 4 °C in the dark. The corresponding working solutions were produced by diluting the stock solutions with deionized water. The tested swine and feed products were purchased from a local supermarket (Tianjin, China) and confirmed to be free of RAC by HPLC-MS/MS analysis.

### 2.2. Instrumentation

A quartz crystal microbalance (QCM 922) from Princeton Applied Research (Seattle, WA, USA) was applied for monitoring the shift in oscillation frequency (Δ*f*, Hz) on an overtone polished 9.0 M Hz AT-cut quartz crystal with Au electrodes on both sides (3000 Å, area: 0.196 cm^2^). A flowing cell (CL_6_) with silicone tubing and a syringe inserted into the mini-fitting were applied to allow the tested solutions to flow over the modified crystal electrode surface. A peristaltic pump (Biosensing Instrument Inc., Tempe, AZ, USA) which could accurately control the liquid flow rate at the range of 20–150 μL min^−1^ was employed to upload the tested solution into the flowing cells at a constant rate. A vortex machine (General Electric Company, Boston, MA, USA) and a solid-phase extraction instrument with 12 parallel channels (Tianjin Agela Technologies Company, Tianjin, China) were used during the preparation process of the chosen swine and feed samples.

### 2.3. Synthesis of RAC Imprinted Material

**Figure 2:**
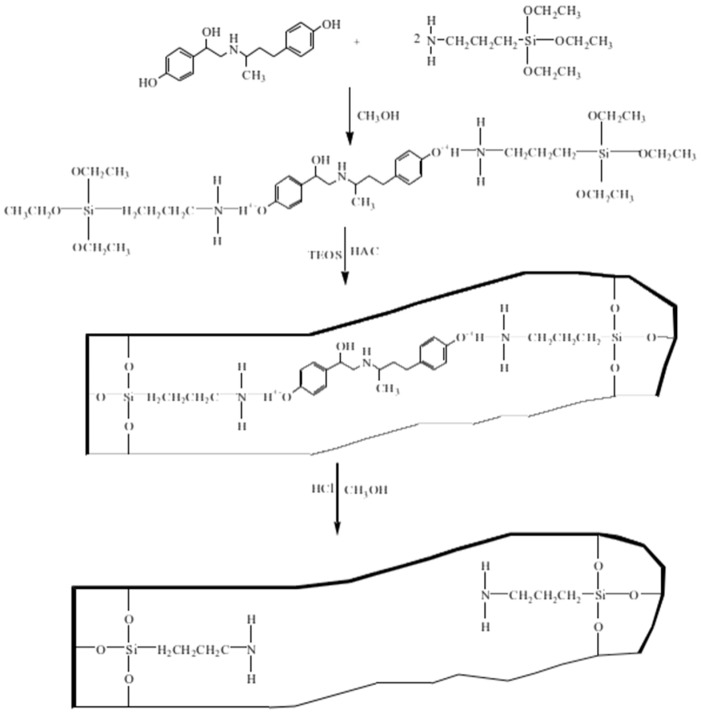
Schematic representation of the synthesis of RAC molecularly imprinted polymer.

The RAC imprinted material used in this study was synthesized in the Key laboratory of Food and Safety, Tianjin University of Science and Technology (Tianjin, China). The template RAC has active phenolic hydroxyl groups and APTES is a basic functional monomer. The N on the amino of APTES can combine with the H^+^ on phenol hydroxyl group of RAC to form the functional silane precursor, and further form the polymer with the cross-linker TEOS. Hydrochloric acid is a strong acid. RAC can be replaced from the polymer and removed by methanol extraction, leaving selective cavies with recognition sites in the polymer (Figure 2). The used RAC imprinted material was verified to have good adsorption capability towards the template RAC (adsorption capacity at an RAC concentration of 120 mg L^−1^: 1.99 mg g^−1^) [12].

Briefly, the synthetic procedure was as follows: accurately weighed RAC (0.5 g) was first dissolved into porogen solvent methanol (3.0 mL) at 40 °C in a glassy tube. The functional monomer (APTES, 1.70 mL) was then added and mixed uniformly. Next, the cross-linker (TEOS, 1.65 mL) was added into the tube and stirred magnetically for 20 min. A catalyzer (HAC solution, 1.0 mol L^−1^, 0.5 mL) was added, and allowed to react for 10 min, and subsequently incubated at 40 °C for 12 h. The solid product was then filtered, washed with methanol, and aged at 100 °C for 10 h. Finally, the polymer product was washed with methanol (35 mL) and HCl solution (1.0 mol L^−1^, 15 mL) to remove the template RAC. The final solid product was dried and used for further research.

### 2.4. Modification of the RAC Imprinted Material on the Au Electrode Surface

Nafion is a commonly used membrane for the immobilization of recognition element in the construction of sensor, through which small ions or molecule can be easily transported. In the research, the Nafion solution was applied for RAC-MIP immobilization on the chip surface. Accurately weighed RAC imprinted material (5.0 mg) was mixed with 100 μL Nafion solution (0.5 wt %) diluted with ethanol in a glass tube. The mixture was then ultrasonically treated to form a homogeneous suspension. The degassing mixture was carefully transferred onto the surface of a QCM Au chip (9 M Hz, AT-cut, 3000 Å, 0.196 cm^2^) fixed in the flowing cell. Finally, the mixture was air-dried in a fume hood, and the whole device was preserved under dry conditions prior to use.

### 2.5. Measurement Procedure for RAC

A series of RAC standard working solutions in the concentrations range 0.5–50.0 mg L^−1^ was prepared. These were then pumped into the flowing cell at a steady rate of 0.1 mL min^−1^ to allow the imprinted polymer binding of the RAC analyte. When the frequency response was stable, a large amount of deionized water was pumped to rinse the MIP-modified chip surface to remove the unbound analyte and attain a constant frequency. The piezoelectric chip was then air-dried in a fume hood and its frequency shift (Δ*f*, Hz) before and after RAC adsorption calculated. Furthermore, an HCl solution (0.1 mol L^−1^) was applied into the flowing cell to desorb the RAC from the imprinted polymer layer on the chip surface. When the frequency response restored to the initial value, deionized water was employed to remove the HCl and the chip was then used for the subsequent RAC analyses. Each measurement procedure can be finished in 8 min. At least three measurements were recorded for each tested RAC concentration. The average Δ*f* value was then used to calculate the change in mass (Δ*m*) on the MIP-modified piezoelectric chip according to the Sauerbrey equation. This was equivalent to the amount of RAC bound to the MIP material.

(1) $$\Delta f=\frac{-2{f_{0}}^{2}\Delta m}{A{\left(\rho_{q}\mu_{q}\right)}^{1/2}}$$

where Δ*f* is the frequency shift, Hz; *f*_0_ is the resonant frequency of the quartz crystal, MHz; Δ*m* is the mass change, g; *ρ*_q_ is the density of quartz (2.65 g cm^−3^), *μ*_q_ is the shear modulus (2.95 × 10^11^ g cm^−1^ s^−2^) and *A* is the piezoelectrically active crystal area (0.196 cm^2^).

### 2.6. Sample Pretreatment and Spiking Method

Swine and feed product samples purchased from a local supermarket were employed to evaluate the potential of the constructed MIP piezoelectric sensor for application as a detector of RAC in food samples. The swine or feed product was accurately weighed (1.0 g) into a glassy tube, and mixed with acetonitrile (10.0 mL) to extract the target analyte and remove any biological impurities. After homogeneous mixing with an oscillator for 5 min, the mixture was centrifuged for 10 min to separate the supernatant. The residue was extracted repeatedly with the same method, and both parts of the supernatant were combined, ultrafiltered (0.22 μm) and dried under N_2_. The product was redissolved in methanol for further research.

To validate the accuracy and precision of the developed MIP piezoelectric sensor for RAC analysis in biological samples, the swine and feed products (1.0 g) were spiked at three levels: 10, 20 and 40 ng RAC, respectively. After treatment using the same procedure, the resulting solution was applied to RAC content analysis using the MIP piezoelectric sensor and HPLC-MS/MS method.

## 3. Results and Discussion

### 3.1. Surface Morphology of the MIP Piezoelectric Sensor

**Figure 3:**
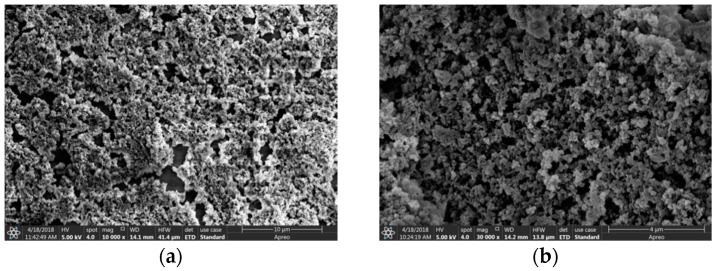
The SEM images of the piezoelectric chip surface modified by RAC imprinted material (**a**) ×10,000; (**b**) ×30,000 magnification.

Scanning electron microscopy (SEM) and atomic force microscope (AFM) were employed to characterize the surface morphology of the developed MIP piezoelectric sensor. The RAC imprinted material covered the chip surface uniformly, and exhibited a porous structure (Figure 3a,b), allowing the recognition sites formed in the cross-linking system to easily bind to the analyte. This result also demonstrated that the Nafion solution could effectively immobilize the MIP material but did not affect its binding properties to the template RAC. From the AFM image (Figure S1A), the thickness of MIP on chip surface was evaluated to be approximate 0.95 μm, and Figure S1B had shown the AFM image of non-edge region of the MIP modified chip surface, from which it can be seen that the MIP is uniformly attached to the chip surface.

### 3.2. Optimization of the Amount of RAC Imprinted Material

In the study, RAC imprinted material was synthesized and used as the recognition element of the piezoelectric sensor for specific binding to RAC. The amount of material modified on the piezoelectric chip surface directly affected the frequency response of the QCM. Thus, the binding amount of RAC was evaluated in detail. Different amounts of RAC imprinted materials (3.0, 4.0, 5.0, 6.0 and 7.0 mg) were individually dispersed in equal volume of Nafion solution (100 μL), and further modified onto piezoelectric chip surface. The frequency response for each sample was then tested under the same experimental conditions.

**Figure 4:**
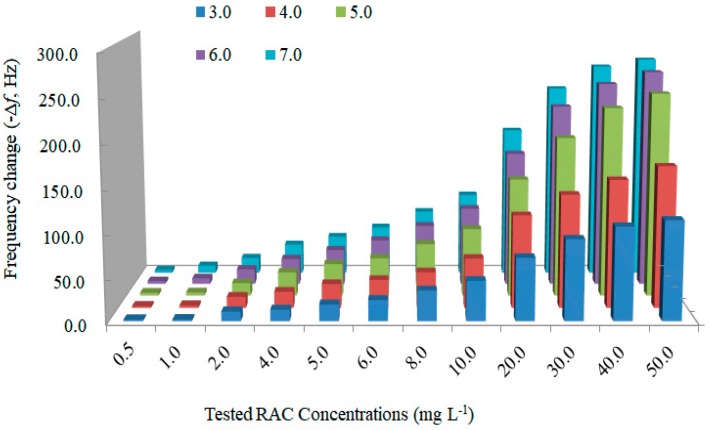
Comparison of the frequency response of the developed piezoelectric chip using different amounts of RAC imprinted material at the concentration range of 0.5–50.0 mg L^−1^.

Figure 4 illustrates that at the same tested RAC concentration, an increase in the imprinted material modified on the chip surface produced a larger frequency response and therefore, more RAC was adsorbed onto the chip surface. Thus, an increase in the tested RAC concentrations (0.5–50.0 mg L^−1^) produced a regular increase in the monitoring frequency response in all tested MIP amounts. When 3.0 and 4.0 mg of imprinted material was applied to the Nafion solution (100 μL), a narrow Δ*f* range (1.8–114.3 Hz and 2.3–164.6 Hz, respectively) of the piezoelectric chip towards the tested RAC concentrations was achieved. The Δ*f* values exhibited wider ranges (3.4–239.9 Hz, 3.5–258.3 Hz, and 3.5–267.6 Hz, respectively) when the amount of RAC imprinted material was increased to 5.0, 6.0 and 7.0 mg, indicating an improvement in the accuracy and precision of the detection process. Additionally, when 6.0 and 7.0 mg of RAC-MIP was added to the Nafion solution (100 μL), the formed membrane appeared to crack and shed off from the chip surface, leading to poor stability and reusability of the developed piezoelectric chip. Based on the above results, 5.0 mg of RAC imprinted material in 100 μL of Nafion solution was established as the optimal amount for MIP modification of the piezoelectric chip.

### 3.3. Response of the Developed Piezoelectric Chip to RAC

Standard working solutions at a series of RAC concentrations (0.5–50.0 mg L^−1^) were individually pumped into a flowing cell in which the RAC–MIP modified piezoelectric chip was first fixed. At least three parallel analyses were conducted for each RAC concentration to establish an average Δ*f* value.

**Figure 5:**
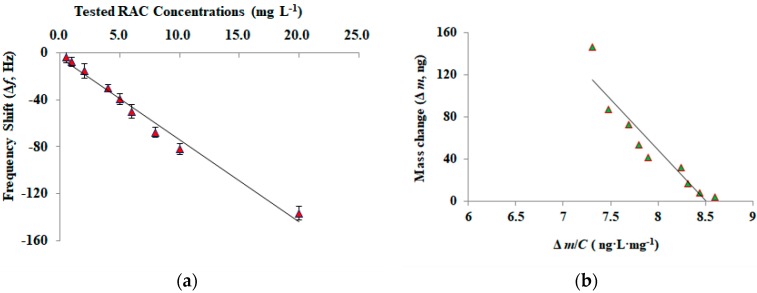
Linear curves of the frequency shift value (Δ*f*) for the tested RAC concentrations (**a**) and Langmuir analysis (**b**).

Figure 5a reveals that with the increase in the tested RAC concentrations resulted in an increase in the Δ*f* value of the piezoelectric chip, signifying that more RAC was bound by the immobilized imprinted material on the chip surface. Good linearity between the Δ*f* value and RAC concentration was observed at the concentration range 0.5–20.0 mg L^−1^ with R^2^ = 0.9845, demonstrating that the system could be applied to the quantitative analysis of RAC. The results revealed limits of detection (LOD, *S*/*N* = 3) and quantification (LOQ, *S*/*N* = 10) for RAC in 0.042 and 0.14 mg L^−1^ standard working solutions, respectively.

Further, the Δ*m* value on the surface of the molecularly imprinted piezoelectric chip at each tested RAC concentration was calculated according to the Δ*f* value. The results were then used to calculate the equilibrium parameters, such as the association constant (*K*_a_) and maximum saturation binding value (Δ*m*_max_) from the Langmuir adsorption isotherm.

(2) $$\Delta m=\frac{\Delta m_{\max}\times K_{a}\times C}{1+K_{a}\times C}$$

After rearranging the equation, the relationship was attained by plotting Δ*m* versus Δ*m*/*C*, with slope 1/*K*_a_ and Y-intercept Δ*m*_max_, as follows:

(3) $$\Delta m=-\frac{1}{K_{a}}\times \frac{\Delta m}{C}+\Delta m_{\max}$$

Thus, Δ*m*_max_ and *K*_a_ were calculated from the plotted data (Figure 5b) as 816.1 ng and 0.0104 mg^−1^ L, respectively, revealing that the synthesized imprinted material displayed a strong affinity toward the RAC analyte.

The selectivity of the developed MIP piezoelectric chip was evaluated using three RAC structural analogues, isoproterenol, terbutaline, and isoxsuprine in the concentration range 0.5–20.0 mg L^−1^ (Figure S2). Notably, out of all the tested analogous, the RAC system afforded the largest Δ*f* values under all the tested concentrations. This was attributed to the better affinity of the imprinted material to the template RAC over that of the other analytes. When the concentration of each analyte was 10.0 mg L^−1^, the selectivity coefficient (Δ*f*_RAC_/Δ*f*_analogue_) for isoproterenol, terbutaline, and isoxsuprine was calculated as 1.96, 1.39 and 1.61, respectively. These results verified that the RAC imprinted material, applied as the recognition element of the piezoelectric sensor, still displayed good selective ability towards the template. The relatively weak Δ*f* value of the analogues is mainly attributed to their similar structure to RAC.

### 3.4. Regeneration of the Developed Piezoelectric Chip

**Figure 6:**
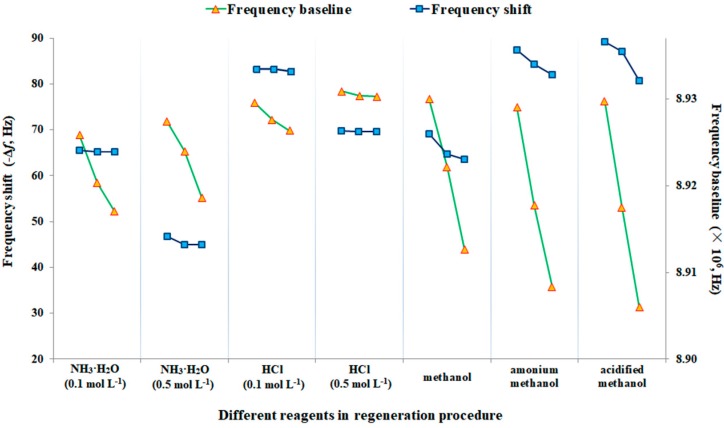
Comparison of the results in the regeneration procedure using different regeneration solutions.

The regeneration process requires the complete removal of RAC from the cavities of the imprinted material. Simultaneously, the polymer layer on the surface of the chip must not be damaged so as not to affect the next determination process. Thus, the conditions for chip regeneration directly affected the accuracy, reproducibility, sensitivity, and lifetime of the developed MIP piezoelectric chip. Based on the previous experiment, a series of solutions with different properties including methanol, water, NH_3_·H_2_O (0.1 and 0.5 mol L^−1^), HCl (0.1 and 0.5 mol L^−1^), ammonium methanol (containing 5% NH_3_·H_2_O), and acidified methanol (containing 5% HCl) were tested for chip regeneration (Figure 6).

No change in the frequency response of the piezoelectric chip (not shown in Figure 6) was observed when pure water was applied for RAC desorption from the MIP material. This suggests that pure water is not effective for RAC removal from the imprinted material. On the other hand, significant frequency shifts were observed when the regeneration solution was acidic or alkaline (HCl and NH_3_·H_2_O, respectively). This occurred because the binding of the imprinted material to RAC is based on the interaction of an acid-base proton pair. Moreover, when the regeneration solution contained methanol, the value of the baseline frequency grew rapidly, signifying that the immobilized MIP layer on the piezoelectric chip surface was damaged, thereby affecting the next analytical process. This was attributed to the dissolution of methanol in the Nafion film causing the MIP material to fall off the surface of the chip. The frequency response returned to the original value when HCl and NH_3_·H_2_O solutions were employed. After three consecutive injection/analysis/recovery processes of an identical RAC solution, the HCl solution (0.5 mol L^−1^) displayed a lower frequency decline (95.1 Hz) than the other regeneration solutions (0.1 mol L^−1^ HCl: 292.7 Hz; 0.1 mol L^−1^ NH_3_·H_2_O: 174.6 Hz; 0.5 mol L^−1^ NH_3_·H_2_O: 965.0 Hz), indicating less damage from the regeneration solution to the immobilized MIP layer on the chip surface. Thus, the 0.5 mol L^−1^ HCl solution was selected for chip regeneration. A single MIP piezoelectric chip could be reused 30 times with a frequency response of 86.5%. After being stored in air for one month, the MIP piezoelectric chip exhibited a frequency response of 94.3%, verifying that the proposed MIP piezoelectric chip displays remarkable reusability and stability for RAC analysis.

### 3.5. Sample Matrix Effect and Recovery Study

Biological impurities, such as proteins and lipids in the swine and feed samples might lead to non-specific adsorption on the MIP modified piezoelectric chip, affecting the accuracy and precision of RAC analysis. In this study, the matrix effect from the selected samples to be removed was evaluated in detail to ensure the accuracy and precision of the developed MIP piezoelectric sensor in RAC analysis.

The matrix standard curves under a series of RAC concentrations (2.5–100.0 μg kg^−1^) in the swine and feed samples are presented in Figure S3. Good linearity (R^2^ ≥ 0.99) was achieved for the two chosen samples with LOD values (S/N = 3) of 0.46 ng g^−1^ (swine) and 0.38 ng g^−1^ (feed). In the spiked and recovery studies, good recoveries at three spiked levels (10.0, 20.0 and 40.0 ng g^−1^) were in the ranges of 75.9–92.1% (swine) and 84.3–93.3% (feed) with acceptable precision values (RSD, n = 3) of 2.3–6.4% (swine) and 3.2–4.1% (feed), respectively (Table 1). The typical HPLC-MS/MS method was also employed for RAC analysis in the spiked samples at each level with recovery results of 78.9–93.0% and an RSD (n = 3) range of 1.8–4.2%. The regression equation for the recovery data from the developed MIP piezoelectric chip and HPLC-MS/MS method was Y = 0.8388X + 12.996 with a correlation coefficient of 0.9611, demonstrating that the developed MIP piezoelectric chip can be applied to accurately detect RAC in swine and feed samples. Table 2 showed the merits of MIP piezoelectric chip for RAC analysis in various samples compared with other methods, indicating the developed MIP piezoelectric chip offered a sensitive, cost-effective and portable method for RAC analysis in complex samples.

## 4. Conclusions

A reproducible molecularly imprinted piezoelectric sensor that allows for the accurate, sensitive and quantitative detection of RAC in swine and feed samples has been successfully developed. The synthesized RAC imprinted material, which is directly immobilized on the piezoelectric chip as the recognition element, exhibits remarkable binding affinity to the target RAC. The developed piezoelectric sensing chip is portable, can be reused for at least 30 times and easily stored for a long time, suggesting its potential value in the on-site, reproducible, and realize low-cost determination of contaminants in food products. This research has promoted further integration of molecular imprinting and piezoelectric sensing techniques, and expanded their application to the development of new strategies and devices in the field of food safety.

## Figures and Tables

**Table 1:** Recoveries of RAC in spiked swine and feed samples using the MIP piezoelectric chip and HPLC-MS/MS.

Sample	Spiked Levels (ng g^−1^)	The Developed MIP Piezoelectric Chip		HPLC-MS/MS	
		Recovery (%)	RSD (%, *n* = 3)	Recovery (%)	RSD (%, *n* = 3)
Swine	10.0	75.9	6.4	78.9	4.2
	20.0	85.6	4.4	84.9	2.7
	40.0	92.1	2.3	93.0	2.4
Feed	10.0	84.3	4.1	86.7	3.7
	20.0	88.4	3.8	89.2	2.6
	40.0	93.3	3.2	92.9	1.8

**Table 2:** Comparison of different methods for RAC determination in various samples.

Methods	Linear Range	LOD	Required Time	Samples	References
**MIP-SPE HPLC**	0.04–18 µg L^−1^	4.6 ng L^−1^	>30 min	Swine	[12]
**Fe_3_O_4_@MIP-SPE HPLC**	0.5–100.0 µg kg^−1^	0.05 µg kg^−1^	>50 min	Swine	[13]
**LC-MS/MS**	—	1.0 µg kg^−1^	>30 min	Bovine muscle	[14]
	0–100.0 µg kg^−1^	1.91 µg kg^−1^	>60 min	Meat, bone meal	[16]
	2.5–20 µg kg^−1^	1.5 µg kg^−1^	4.5 min	Swine	[17]
**GC/MS**	10.0–500.0 µg L^−1^	4.0 µg kg^−1^	>60 min	Feed	[15]
**Fluorescence immunoassay**	2.3–50 µg L^−1^	1.0 µg L^−1^	10 min	Turkey meat	[18]
**Electrochemical immunosensor**	0.01–5 µg∙mL^−1^	2.3 ng L^−1^	>2 h	Swine urine	[19]
	1–40 µg L^−1^	0.34 µg L^−1^	—	Swine muscle	[21]
**Electrochemical aptasensor**	10^−12^–10^−8^ mol L^−1^	5.0 × 10^−13^ mol L^−1^	>35 min	Swine urine	[20]
**MIP piezoelectric sensor**	2.5–100.0 μg kg^−1^	0.46 and 0.38 ng g^−1^	<8 min (reused at least 30 times)	Swine, feeds	This research

## Supplementary Materials

The following are available online at http://www.mdpi.com/1424-8220/18/6/1870/s1, Figure S1: AFM image of the edge (A) and non-edge (B) region of RAC-MIP modified chip, Figure S2: Response of the developed MIP piezoelectric chip to RAC and its structural analogues, Figure S3: The matrix standard curves under the RAC concentrations of 2.5–100.0 μg kg^–1^ in the swine and feed products.
