# Supplementary figures and images for: Reproducible Molecularly Imprinted Piezoelectric Sensor for Accurate and Sensitive Detection of Ractopamine in Swine and Feed Products
Source: Sensors (Basel). 2018 Jun 7;18(6):1870. doi: 10.3390/s18061870 (PMC6022169; doi:10.3390/s18061870)

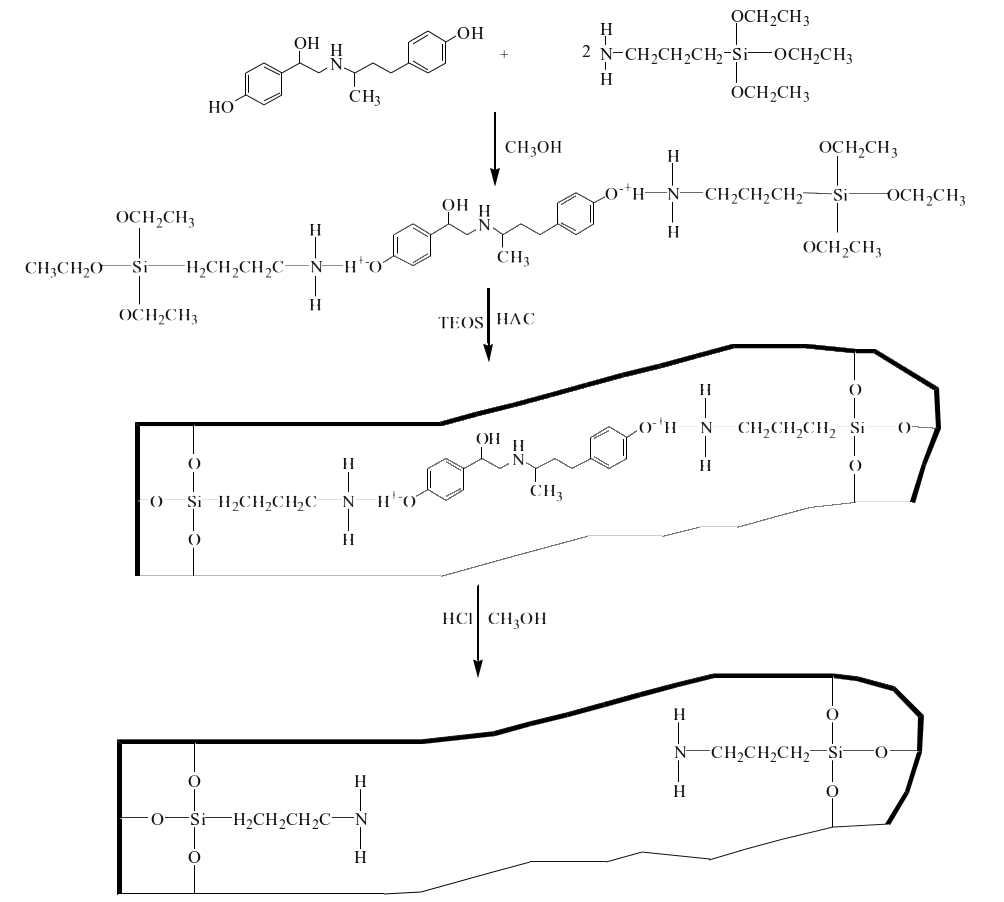

Supplement: Supplementary file 1 [file sensors-18-01870-s001.zip › Figures/Figure 2.tif]

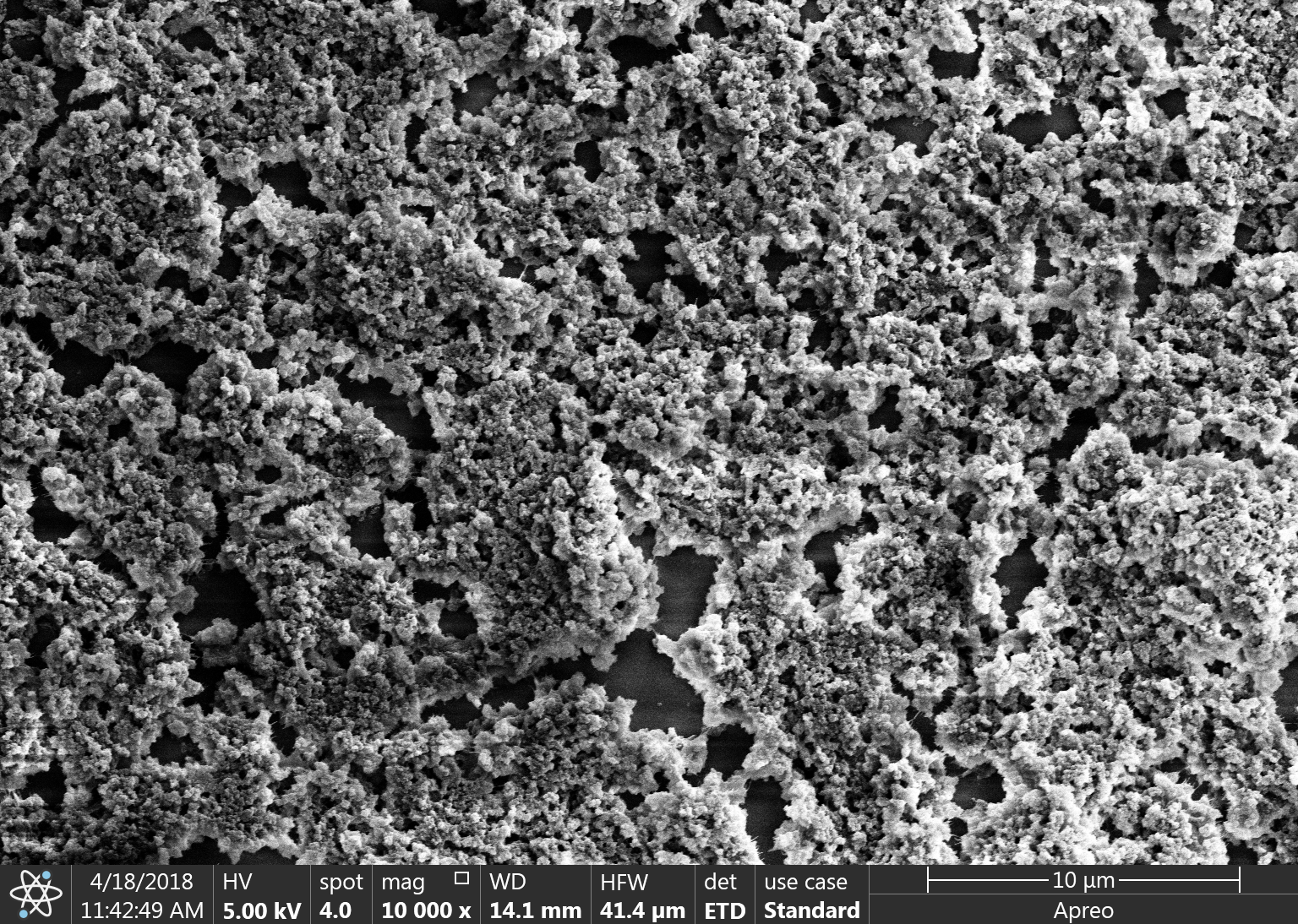

Supplement: Supplementary file 1 [file sensors-18-01870-s001.zip › Figures/Figure 3 a.tif]

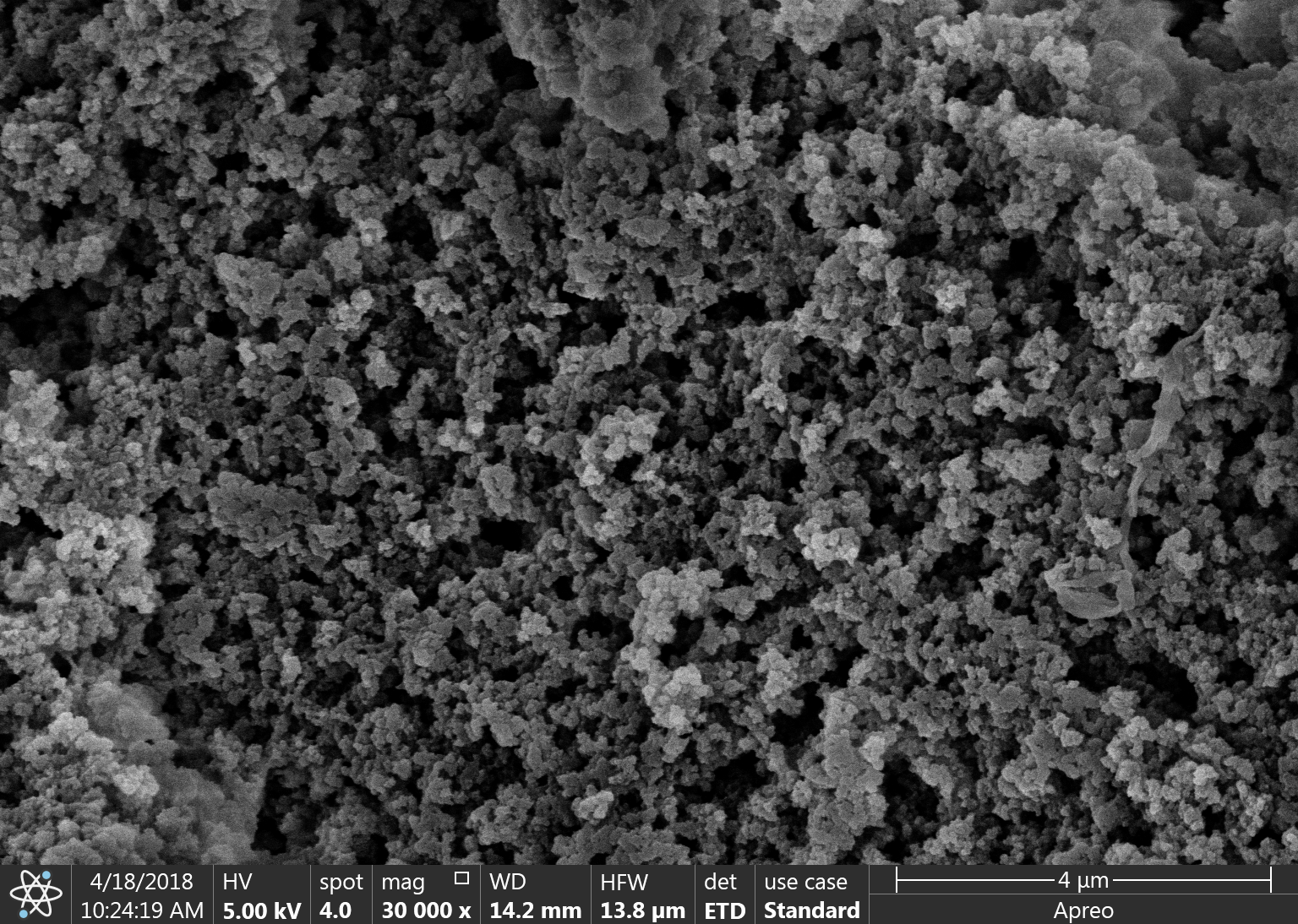

Supplement: Supplementary file 1 [file sensors-18-01870-s001.zip › Figures/Figure 3 b.tif]

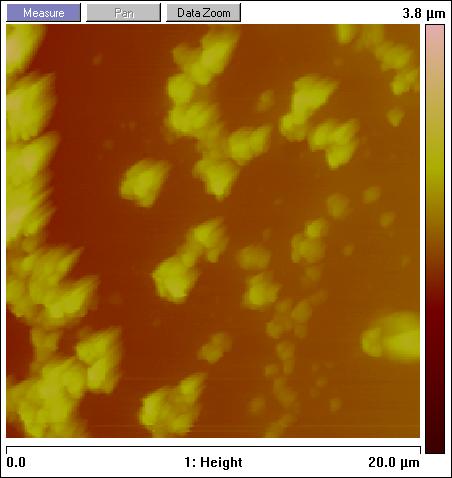

Supplement: Supplementary file 1 [file sensors-18-01870-s001.zip › Figures/Figure 3 c.jpg]

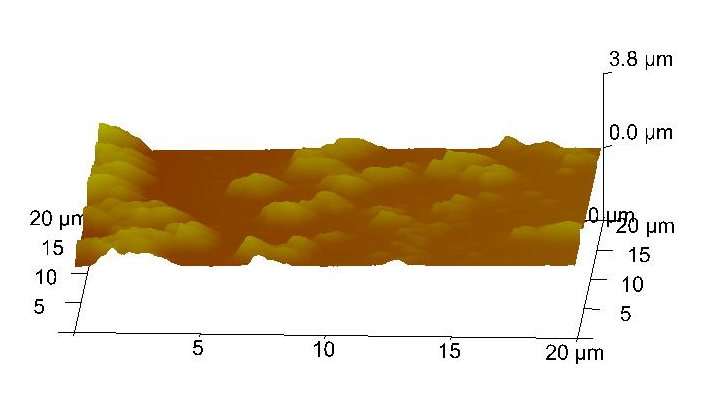

Supplement: Supplementary file 1 [file sensors-18-01870-s001.zip › Figures/Figure 3 d.tif]

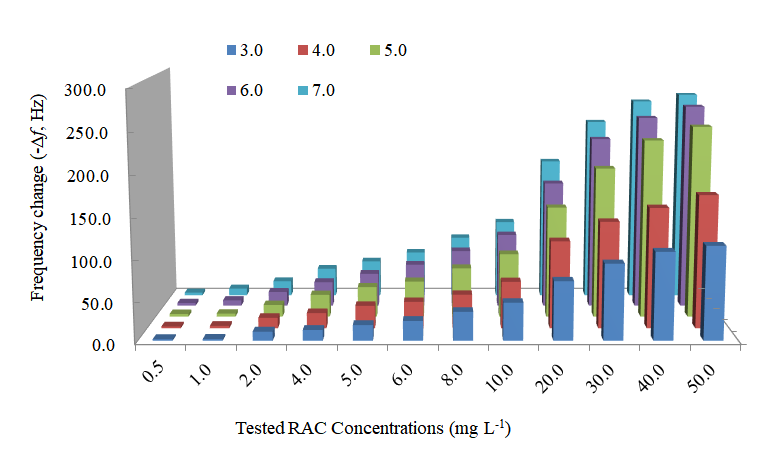

Supplement: Supplementary file 1 [file sensors-18-01870-s001.zip › Figures/Figure 4.tif]

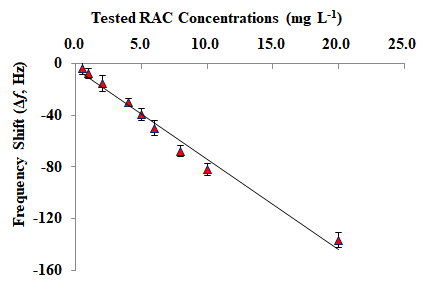

Supplement: Supplementary file 1 [file sensors-18-01870-s001.zip › Figures/Figure 5 a.tif]

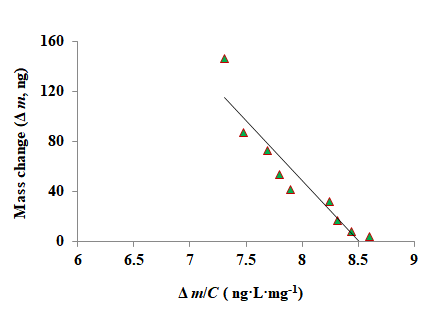

Supplement: Supplementary file 1 [file sensors-18-01870-s001.zip › Figures/Figure 5 b.tif]

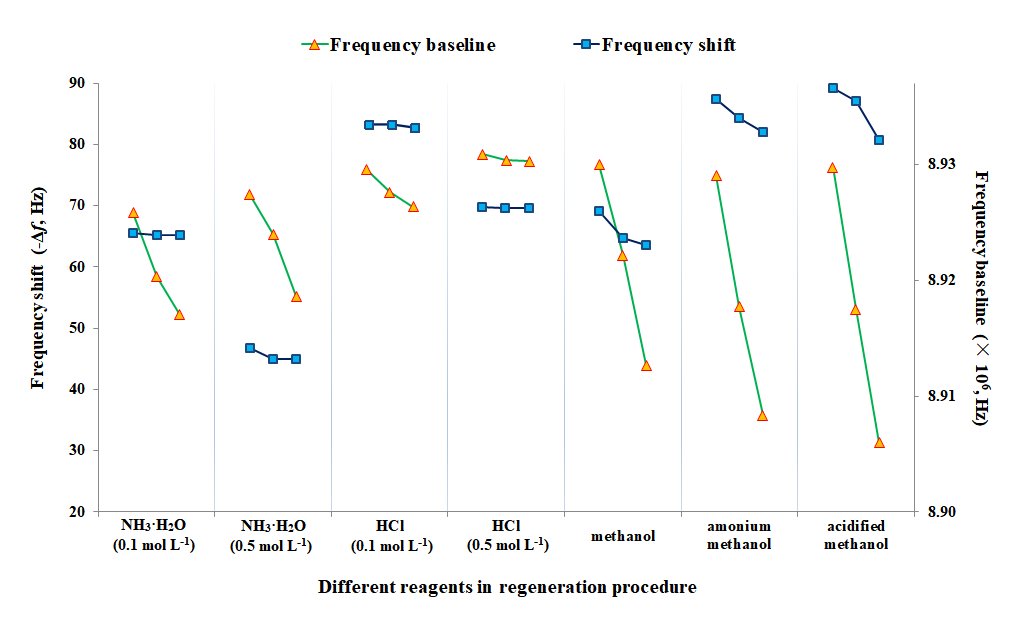

Supplement: Supplementary file 1 [file sensors-18-01870-s001.zip › Figures/Figure 6.tif]
